# Supplementary material for: Evaluation of Anesthetic Specific EEG Dynamics during State Transitions between Loss and Return of Responsiveness
Source: Brain Sci. 2021 Dec 28;12(1):37. doi: 10.3390/brainsci12010037 (PMC8773581; doi:10.3390/brainsci12010037)
Supplement: Supplementary file 1 [file brainsci-12-00037-s001.zip › brainsci-1503037-supplementary.pdf]

Supplemental Information for *Evaluation of Anesthetic Specific EEG Dynamics during State Transitions between Loss and Return of Responsiveness*

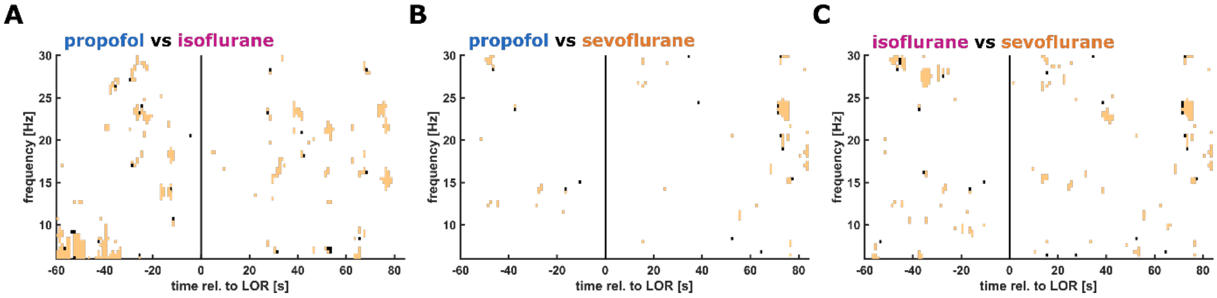

Figure S1. AUC matrices derived from the comparisons of the EEG during propofol-induced LOR between the different maintenance groups. The dots indicate a significant difference in that frequency at that time-point. Because every frequency at each time point was compared, only large clustered areas of significant differences should be considered. These areas cannot be observed here, i.e. there were no clusters of significant differences when comparing the propofol-induced LOR of A. the propofol maintenance group vs. the isoflurane maintenance group; B. the propofol maintenance group vs. the sevoflurane maintenance group; C. the isoflurane maintenance group vs. the sevoflurane maintenance group.

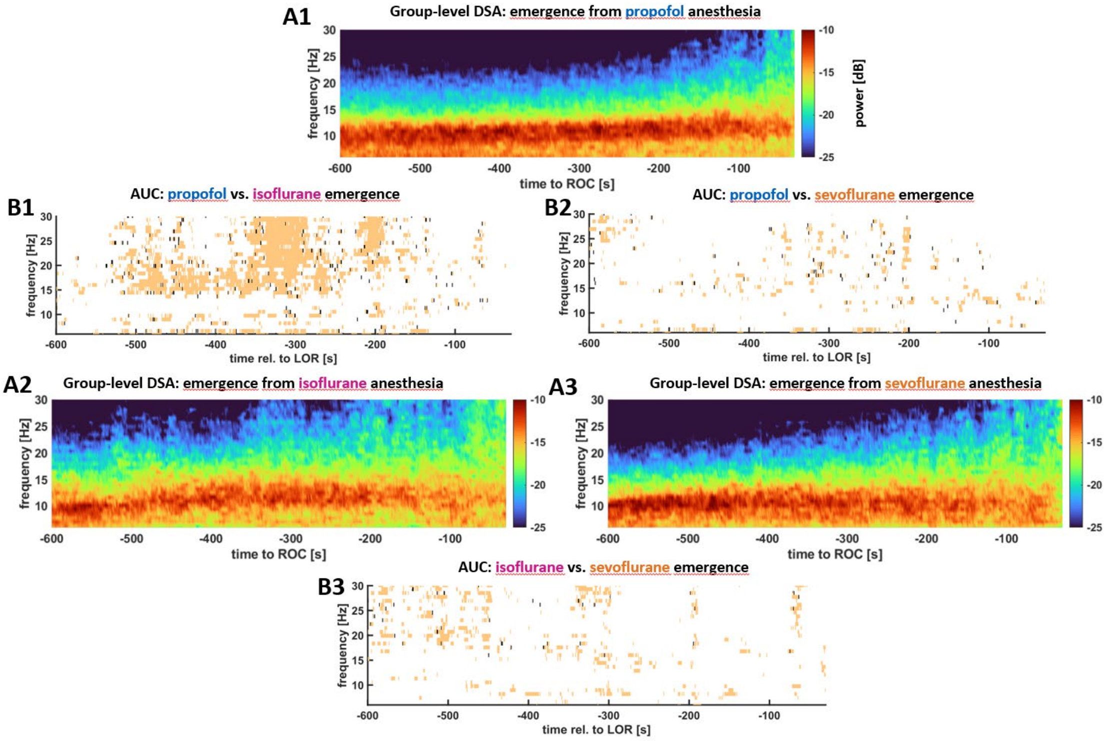

Figure S2. Density spectral arrays (relative power) plots for all three groups derived from the pooled (median) relative power spectral density of all patients within a group (**A**) and the corresponding information regarding a significant difference (**B**) as determined by the 95% confidence intervals of the AUC. **A**. DSA of anesthesia emergence for the different substance groups. **A1**. Group-level emergence DSA of the patients receiving propofol. **A2**. Group-level emergence DSA of the patients receiving isoflurane. **A3**. Group-level emergence DSA of the patients receiving sevoflurane. **B**. Maps of the pixel-wise AUC calculations. **B1**. Comparison between the propofol and isoflurane group. **B2**. Comparison between the propofol and sevoflurane group. **B3**. Comparison between the isoflurane and sevoflurane group. During emergence from propofol maintenance, the power in the frequencies above ~15 Hz was significantly lower than from maintenance with isoflurane (**B1**). There were no significant differences in the emergence behavior between propofol and sevoflurane (**B2**), and sevoflurane and isoflurane (**B3**).

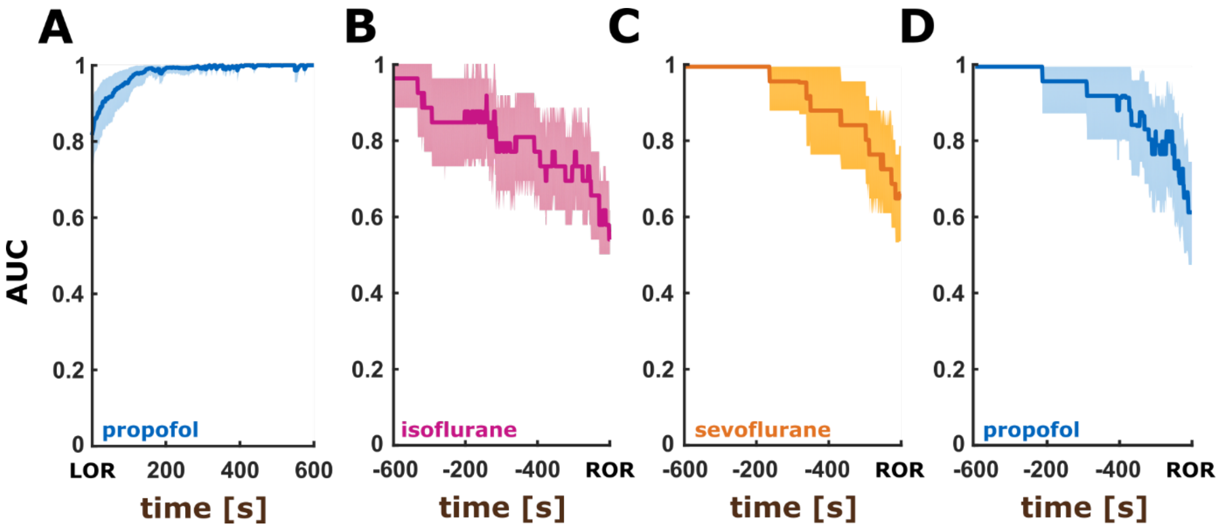

Figure S3. Association between index performances based on AUC analysis for the EMG index over the course of 600 s. The trends show the AUC and corresponding 95% confidence intervals for the comparison of the EMG values at the time on the *x*-axis compared to the EMG values at loss of responsiveness (**A**) or return of responsiveness (ROR, **B**–**D**). (**A**) At LOR, the AUC curve showed a strong and fast change of EMG activity after LOR, indicative of decreasing EMG activity due to anesthesia induction. (**B**–**D**) For all maintenance anesthetic regimen (**B**) isoflurane, (**C**) sevoflurane, and propofol a strong change of the EMG index throughout the emergence period could be observed.

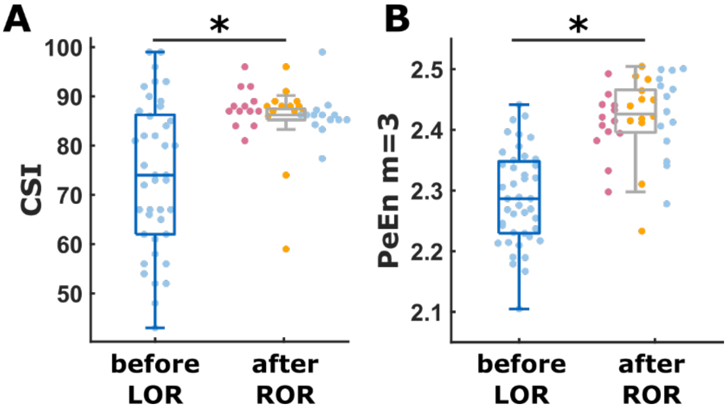

Figure S4. Difference in CSI (**A**) and PeEn (**B**) before LOR (LOR-15s) and after ROR (ROR+30s), i.e., at time points the patients were responsive. The parameter values after ROR were significantly (*) higher ($p < 0.001$). A. There was a significant difference between the CSI values before LOR and after ROR; B. There was a significant difference between the PeEn values before LOR and after ROR.
